# Post-Consumer Carpet Fibers in Concrete: Fiber Behavior in Alkaline Environments and Concrete Durability

**DOI:** 10.3390/ma17050977

**Published:** 2024-02-20

**Authors:** Aswathy Simon, Barzin Mobasher, Narayanan Neithalath

**Affiliations:** School of Sustainable Engineering and Built Environment, Arizona State University, Tempe, AZ 85287, USA

**Keywords:** post-consumer carpet fibers, alkaline exposure, recycling, concrete, durability

## Abstract

The widespread use of carpets in residential and commercial buildings and their relatively short life span result in large volumes of carpet being landfilled. A potential solution to this problem is the use of post-consumer carpet fibers in concrete. To this end, this paper systematically identifies the common fiber types in a typical post-consumer carpet fiber bale and evaluates their durability under exposure to varying levels of alkalinity. The tensile strengths and toughness of the fibers belonging to the nylon and polyethylene terephthalate (PET) families (the dominant fibers in most post-consumer carpets) are reduced by up to 50% following exposure to extreme alkalinity, the reasons for which are determined using spectroscopic and microscopic evaluations. The chloride ion transport resistance of concretes (~40 MPa strength) containing 2.5% carpet fibers by volume (~25 kg of fibers per cubic meter of concrete) is comparable to that of the control mixture, while mortar mixtures containing the same volume fraction of carpet fibers demonstrate negligible enhancement in expansion and loss of strength when exposed to 1 N NaOH. This study shows that moderate-strength concretes (~40 MPa) for conventional building and infrastructure applications can be proportioned using the chosen volume of carpet fibers without an appreciable loss of performance. Consideration of low volume fractions of carpet fibers in low-to-moderate-strength concretes thus provides a sustainable avenue for the use of these otherwise landfilled materials in construction applications.

## 1. Introduction

Most residential and commercial buildings in many parts of the world use carpets as the preferred floor-covering material [1]. In general, the carpet industry uses new fibers to produce carpets, with millions of tons of fibers being produced and consumed annually [2,3]. In the meantime, a large quantity of carpets are replaced annually, and the discarded ones are disposed of in landfills, primarily because the reprocessing and reuse of carpets is difficult and expensive. It is stated that only about 5% of discarded carpet is actually recycled in the U.S., and only 1% is recycled into new carpets [4]. According to some estimates, ~4% of the volume of solid wastes disposed of at landfills in the U.S comprise of discarded carpets [5], amounting to more than two million metric tons every year. The high volume-to-weight ratio and increasing carpet consumption also poses questions in terms of landfill availability. Approximately 50% of the volume of a carpet is made of face fibers [6], which are generally made from plastics sourced from petrochemicals, and hence take a very long time to decompose. Moreover, their decomposition releases methane, a potent greenhouse gas, along with a host of other toxic chemicals into the environment [7]. Hence, beneficial utilization of post-consumer carpets, or recycling them into newer carpets, is urgently needed [1]. In 2010, the State of California implemented the Carpet America Recovery Effort (CARE) [8] to enable increased use of recycled carpets and waste carpets in other beneficial applications. 

The face fibers in carpets are generally constituted of synthetic materials such as nylon, polypropylene, polyester, polyethylene terephthalate (PET), and mixed synthetics or natural fibers such as wool, and could be beneficially used if properly collected and sorted [1,2,6,9,10]. The surface fibers can be removed from carpets by simple shredding, cutting, and shearing techniques, or through the use of sophisticated machinery [2,10]. While nylon and PET fibers (which form a significant fraction of disposed carpet fibers) can be recycled, it is important to note that only nylon 6 is currently economically recyclable back into face fiber [11]. This is one prominent reason for a lot of post-consumer carpets ending up in landfills. Recycled carpets are used in sound insulation products, tiles, plastic pellets, furniture manufacturing, and sometimes as a fuel in cement kilns [6,12,13,14]. The latest estimates show that about 6% of the U.S.’s carpet waste is burned in conventional municipal solid waste incinerators and cement kilns [4], creating significant air pollution and health hazards. 

Synthetic fibers similar to those found in carpets have been used in concrete to enhance their early- and later-age resistance to volume changes and cracking, and sometimes to improve strength and ductility. Past studies have evaluated the use of recycled carpet fibers in concrete [1,15,16], either by separating the different classes of fibers or using them as-extracted from carpet wastes. Our previous work [17] evaluated the use of carpet fibers from shredded carpets obtained from landfill operators in California, without separating the individual fiber types in the carpet in an attempt to keep the cost of using recovered carpets in concrete low. Fundamental characterization of the fiber types in the carpet waste, methodologies used to disperse these fibers in concrete, and the development of concrete mixture design guidelines for low-to-moderate-strength (≤40 MPa) applications using such fibers were elucidated in detail. The fiber volumes in the concrete mixtures were optimized in our study such that the desired 28-day strengths could be achieved without significantly increasing the cement content or reducing the water-to-binder ratio, both of which increase the cost of concrete and detrimentally affect commercial uptake. A cradle-to-gate life cycle analysis (LCA) demonstrated that concretes containing 2.5% carpet fibers by volume and a cement replacement of 20% (by mass) with Class F fly ash resulted in such concretes performing better than conventional concretes of similar strength in all the environmental impact categories [17]. 

In order to ensure that sustainable concretes incorporating post-consumer carpet fibers are adopted for use in buildings and other structures, especially in regions where carpet waste collectors and landfill operators are proximal, it is important to ensure that the long-term performance of such concretes is also guaranteed. This is especially important since carpet fibers have a propensity for high water absorption and/or to depolymerize and deteriorate in the alkaline environment in concrete. While past studies have reported the mechanical properties of concretes containing carpet fibers, there has been little work elucidating the chemistry of different fiber types in a typical carpet bale and their degradation characteristics, along with economical, sustainable, and durable concretes that employ these fibers. This paper therefore reports detailed studies on the changes in the mechanical properties and microscopic and spectroscopic characteristics of the fibers under exposure to alkaline environments commonly encountered in concrete, along with the volume change and strength loss of mortars and the ionic transport performance of concretes [18,19]. Post-consumer carpets that are landfilled contain different types of face fibers, with nylon and PET fibers being the most common ones. This study hence uses fibers from a representative bale containing ~90% nylon and PET fibers, and uses waste carpet bundles containing such fiber type distributions in the concretes that are proportioned and tested. The results from these comprehensive studies can be used in devising appropriate standards for the use of carpet fibers in moderate-strength concrete (herein defined as ≤ 40 MPa) for many building applications such as slabs on grade (both reinforced and unreinforced), walls, and other structural members.

## 2. Experimental Program

### 2.1. Materials 

**Figure 1:**
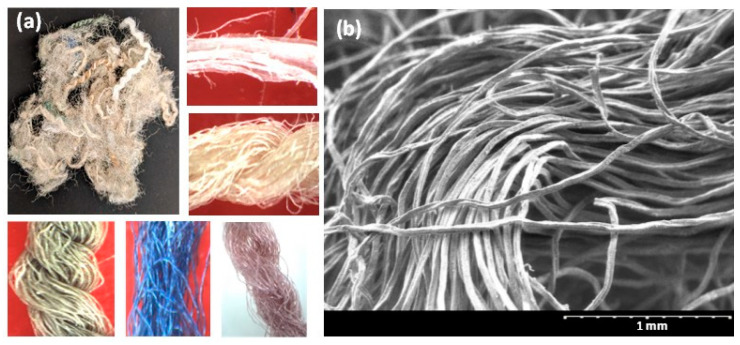
(**a**) A random collection of fibers from the carpet fiber bundle along with magnified optical images of individual fibers (note that each fiber shown here is composed of numerous strands) and (**b**) electron micrograph of a typical CF showing the strands.

Fibers: CMJ Systems Inc. (Phoenix, AZ, USA) supplied the post-consumer carpet fibers (CFs) procured from landfill operators in California. The carpet fibers, obtained by sorting and shredding carpets removed from existing buildings, were heterogeneous in length, diameter, chemistry, and physical properties (density, water absorption, etc.). The CFs procured for this work contained fibers with lengths ranging from 20 mm to 55 mm and diameters ranging from 0.5 mm to 2.0 mm (each fiber contained several strands with diameters ranging from 10 to 50 μm, as shown in the micrographs—see Figure 1). The aspect ratio of CFs ranged from 22 to 100. Figure 1 shows optical images and electron micrographs of individual CFs (single/multi-strands) randomly collected from the supplied fiber mass. The average specific gravity of the fibers was found to be 0.95, as measured using a pycnometer. 

**Figure 2:**
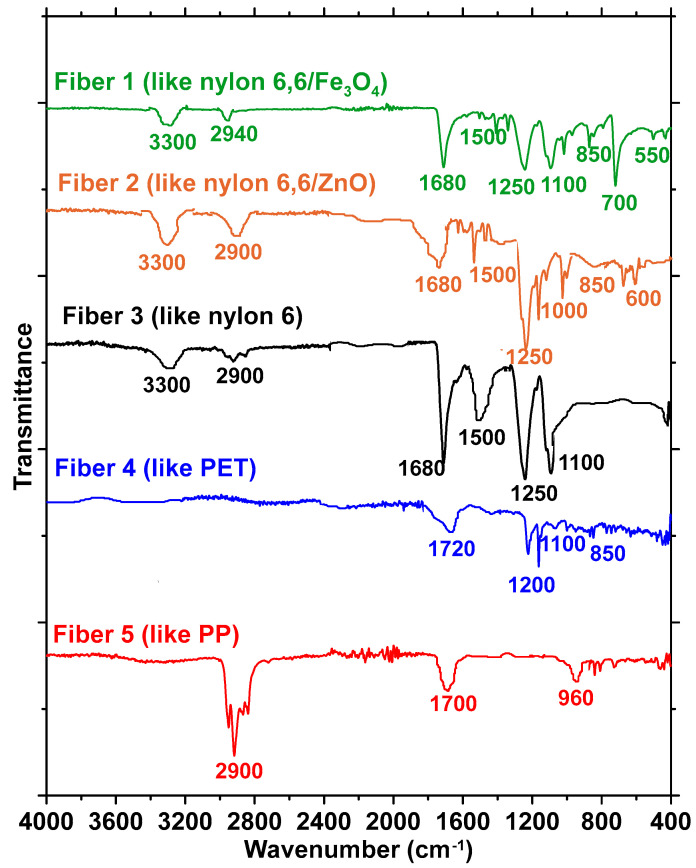
FTIR spectra of the five main classes of fibers present in the fiber bundle. Fibers 1, 2, and 3 belong to the nylon family, while Fiber 4 is similar to PET and Fiber 5 is similar to PP.

As shown in Figure 1, CFs are an amalgamation of different types of fibers. As per the recycling contractor, the major constituents in carpets that are being discarded in landfills (i.e., not recycled for either new carpets or other applications) were nylon and polyethylene terephthalate (PET), as identified using near-infrared spectroscopy in a bulk handling process. To ascertain this, several strands from different bundles of CFs were subjected to Fourier transform infrared (FTIR) spectroscopy in the attenuated total reflectance (ATR) mode using a diamond ATR crystal. The spectra thus obtained for these fibers were found to fall into five distinct categories, as shown in Figure 2, with three belonging to the nylon family and the remaining two being PET and polypropylene (PP), respectively. For PET fibers, prominent peaks appear in the FTIR spectra shown in Figure 2 at wavenumbers of 1720, 1200, 1100, and 850 cm^−1^, corresponding to C=O, C(=O)-O, and C-H bonds, respectively [20]. For PP, the peaks at 2900, 1700, and 960 cm^−1^ correspond to N-H and C=O bond vibrations [21], while for nylon, the peaks at 3300, 2900, 1680, and 1500 cm^−1^ are generally attributed to N-H stretching, C-H symmetric and asymmetric stretching, C=O stretching, and N-H bending, respectively [22]. FTIR analysis of 100 s conducted on fibers from the bundle revealed that most of the fibers are constituted of PET, in line with what was indicated by the supplier. A quantification exercise revealed that around 50% of the fibers within the as-provided fiber bundles were PET, while approximately 40% belonged to the nylon family. The remaining 10% were PP fibers. Among the fibers belonging to the nylon family, three different spectra were identified—one corresponding to nylon 6 and two to nylon containing metal oxides (nylon 6,6/Fe_3_O_4_ and nylon 6,6/ZnO); in this case, the intensity of amide A (wavenumbers 3300–3500 cm^−1^) and amide I (wavenumbers 1600–1800 cm^−1^) bands differentiate the spectra [23,24]. More details on fiber characterization, including those based on fiber strength, modulus, and elongation, can be found in our recent work [17]. 

### 2.2. Mixture Proportions 

Mortars and concrete: The binder phase of the mortars and concretes used in this work consisted of Type I/II ordinary Portland cement (OPC) conforming to ASTM C 150 [25] and a typical Class F fly ash conforming to ASTM C 618 [26]. The chemical composition and physical properties of OPC and fly ash are listed in Table 1. The fine aggregate used in this study was a mixture of equal quantities of commercially available medium and coarse sands with particle sizes ranging from 0.3 to 0.8 mm and 0.6 to 1.7 mm, respectively. The coarse aggregates consisted of particles with a nominal maximum size of 12.5 mm. A commercial polycarboxylic ester (PCE) admixture was used for the mortar and concrete mixtures. 

The mortar mixtures used in this work included control mixtures with and without fly ash, and mixtures containing 2.5% or 5% of fibers by volume. The water-to-binder ratio (w/b) used was 0.41 or 0.45 based on the fiber volume fraction used. Fibers from the as-provided carpet bale (which is representative of the type of carpets handled by the post-consumer carpet waste landfill operator) were used; the carpet bale contains all the types of fibers identified as mentioned earlier, with dominant proportions of nylon and PET. The mortar and concrete proportions were arrived at based on previous work [17], which deals extensively with proportioning aspects and LCA of concretes with different volume fractions of CFs. The mortar mixture proportions shown in Table 2 were used for experiments to determine dimensional stability and strength loss. The concrete mixtures, shown in Table 3, were proportioned for chloride ion transport studies and contained only 2.5% fibers by volume since a larger fiber volume resulted in difficulties in dispersing the fibers uniformly and mixing them in concrete to obtain a cohesive and homogeneous mixture. The need to adequately distribute CF fibers that are composed of a multitude of strands and the relatively high moisture absorption levels of the fiber bundle necessitated a modified mixture-proportioning procedure for CF-reinforced mortars and concrete, which have been explained in detail elsewhere [17]. In short, the fibers were separated into individual strands from the bundle through hand separation (in an industrial setting, a blower can be used before introducing fibers into the mixer, as was carried out by our industrial partner for a slab-on-grade construction, which was the basis for further certification tests for CF-reinforced concretes for commercial use derived from this research), followed by the addition of sand (and coarse aggregates, for concrete), along with a part of the mixing water and admixtures and a quarter of the fibers. Following uniform mixing, fibers were then added in 25% increments along with the corresponding amount of water and admixtures and mixed. Finally, cement, fly ash, and the remaining water were added and mixed for 5 min until the desired consistency was achieved. While this mixing procedure worked consistently for the studies reported here, minor changes would likely be required for other studies elsewhere based on the widely varying CF characteristics. 

For dimensional stability tests, mortar beams (25 mm × 25 mm × 285 mm) were cast, while for compressive strength tests, mortar cubes (50 mm × 50 mm × 50 mm) were used. For the chloride transport tests (rapid chloride permeability (RCP) and non-steady state migration (NSSM)), cylinders 100 mm × 200 mm in size were cast using the mixtures shown in Table 3, and 50-mm thick specimens were cut from the cylinders using a water-cooled diamond saw. 

### 2.3. Fiber Exposure to Alkaline Environments and SEM and FTIR Tests

**Figure 3:**
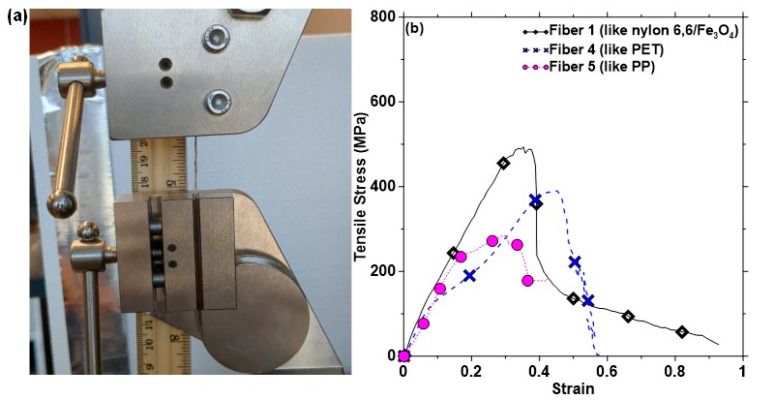
(**a**) An image of a fiber undergoing tensile test and (**b**) representative stress–strain curves of three fibers demonstrating the differences in the peak tensile stresses and strains of fibers belonging to the same fiber bundle.

It is critical to examine the influence of concrete’s alkaline environment on the performance of CFs. All five fiber types identified using FTIR (Figure 2) were immersed in Ca(OH)_2_ solutions designed to obtain three different pH levels (pH 10–13) to simulate different alkaline environments, including the pore solution in concrete (pH of ~12–13). The fibers were also submerged in 1 N NaOH (prepared by dissolving 40 g of NaOH in 1 L of deionized water) to simulate extreme alkaline exposure (e.g., to simulate exposure to highly alkaline pore solutions as in alkali-activated binders). The exposure to these aggressive solutions continued for 30 days. At the end of the treatment duration, the fibers were subjected to FTIR spectroscopy to determine the changes in chemical bonding as a result of alkaline exposure. The fibers were also evaluated, before and after exposure, using high-resolution field-emission scanning electron microscopy (SEM) in the secondary mode under an accelerating voltage of 5 kV. The treated fibers were subjected to uniaxial tension in a 5 kN tension testing machine (MTS EXCEED E42, MTS, Eden Prairie, MN, USA) in displacement-controlled mode, as shown in Figure 3a, and representative stress–strain curves of a few unexposed fibers tested to serve as the control case are shown in Figure 3b. The loading rate was maintained at 5 N/s, and the gage length was maintained at 24 mm. Five to ten samples belonging to each fiber type (based on FTIR analysis) and each exposure condition were tested since the heterogeneous nature of CFs demands a larger sample size. 

### 2.4. Tests on Mortars

The dimensional changes in CF-reinforced mortars exposed to alkaline environments were determined in accordance with ASTM C157 [27]. The mortar beam specimens were immersed in 1 N NaOH solution after three days of moist curing to evaluate the change in length. Companion specimens were cured at >98% RH and 23 ± 2 °C for 28 d. To determine the strength loss under exposure to an alkaline environment, 50 mm mortar cubes were tested in compression. The test conditions selected for the study include the following: (a) cubes cured in water for 7 d and kept in 1 N NaOH for 21 d (total of 28 d) and (b) cubes cured in water for 28 d. The dimensional change and strength loss tests were carried out only under exposure to 1 N NaOH (and not under Ca(OH)_2_ solutions) to simulate the most aggressive case. Compressive strength tests were carried out in accordance with ASTM C109 [28]. 

### 2.5. Chloride Transport Tests

Rapid chloride permeability (RCP) (ASTM C1202 [29]) and non-steady state migration (NSSM) tests (NT BUILD 492 [30]) were conducted on 50 mm thick slices of concrete samples cut from 100 mm × 200 mm cylinders that were cured in a moist environment for 28 and 56 days. RCP test specimens were vacuum-saturated with deionized water and sandwiched between cells containing 0.3 N NaOH solution on one side and 3% NaCl solution on another side. A potential difference of 60 V was applied between the electrodes placed on both faces of the specimen for 6 h. The total charge passed through the sample at the end of 6 h is reported as the RCP value. Even though the RCP test has several drawbacks [31,32,33], it is still accepted as a standard test method for concretes exposed to chloride-containing environments. For the NSSM test, the specimens were vacuum-saturated in a Ca(OH)_2_ solution and enclosed between 2 N NaCl catholyte and 0.3 N NaOH anolyte solutions. A potential difference of 30 V was maintained for 24 h (this potential difference was selected based on the initial current reading). After the test duration, the specimens were axially cut, and 0.1 N AgNO_3_ solution was sprayed on the cut surface. The chloride penetration depth was measured based on the precipitation of white silver nitrate. The non-steady state migration coefficient (DNSSM) in m^2^/s is obtained as

(1) $$D_{NSSM}=\frac{RT}{zFE}\cdot \frac{X_{d}-\alpha \sqrt{X_{d}}}{t}$$

(2) $$\alpha =2\sqrt{\frac{RT}{zFE}}\cdot {\mathrm{erf}}^{-1}\left(1-\frac{2C_{d}}{C_{0}}\right)$$

Here, *E* = (*U* − 2)/*L*, where *L* is the specimen thickness in m, and *U* is the absolute value of the applied voltage in volts. The valence of the chloride ion is represented by *z*, *F* is the Faraday constant, and *R* is the mortar gas constant. *T* is the average value of initial and final temperatures in K. *X_d_* is the average penetration depth in m, *t* is the test’s duration in seconds, *C_d_* is the chloride concentration at which silver nitrate changes to silver chloride, and *C_0_* is the chloride concentration of the catholyte solution (2 N). The value of *C_d_* chosen for this study is the value that is generally reported for OPC concrete (~0.07 N) [34].

## 3. Results and Discussions

### 3.1. Tensile Strength and Toughness of Fibers under Different Exposure Conditions

**Figure 4:**
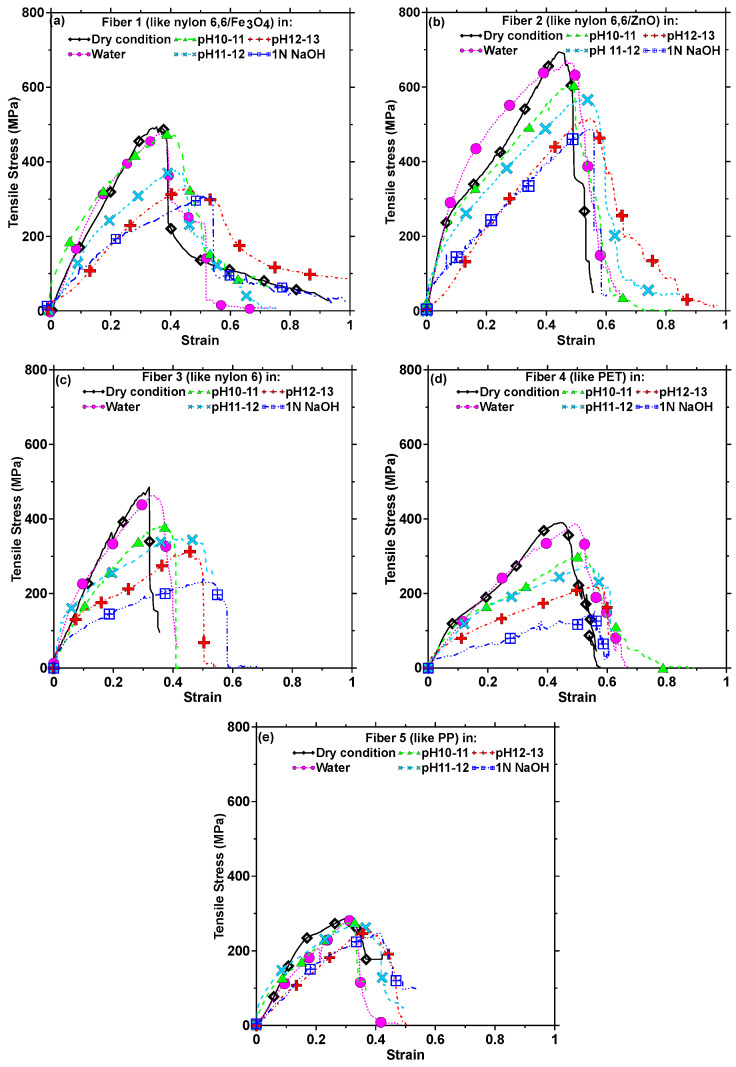
Representative stress–strain curves of selected fibers present in the CF bundle under six different exposure conditions (as-received or dry condition, immersed in water for 24 h, Ca(OH)_2_ solution of pH 10–11 for 30 days, Ca(OH)2 solution of pH 11–12 for 30 days, Ca(OH)_2_ solution of pH 12–13 for 30 days, and 1 N NaOH solution for 30 days). (**a**) Fiber 1, similar to nylon 6,6/Fe_3_O_4_; (**b**) Fiber 2, similar to nylon 6,6/ZnO; (**c**) Fiber 3, similar to nylon 6; (**d**) Fiber 4, similar to PET; and (**e**) Fiber 5, similar to PP.

The stress–strain curves of the different classes of fibers in the CF bundle in the as-provided state (i.e., without exposing them to water or any alkaline solutions) are shown Figure 4 (along with the results of those exposed to different conditions). Tests were carried out on five or more representative samples of each of the fiber types in the carpet bundle (see Figure 3) to ensure that the changes in tensile response under an alkaline environment are adequately captured. Table 4 lists the reported virgin tensile properties of different fiber types observed in the CF samples. The average tensile strengths obtained from experiments for Fibers 1, 2, and 3 in the dry state are ~500–700 MPa, which conform to the tensile strength values of fibers belonging to the nylon family [35,36]. The elongation at peak load is in the range of 30% to 40% for these fibers. The average tensile strength of Fiber 4 is 391 MPa, and its elongation at peak load is about 45%; these values are in line with those of polyester fibers (the most common polyester used in the carpet industry is PET) [37,38]. For Fiber 5, the average tensile strength is found to be ~285 MPa and the elongation at peak load is ~30%, which are similar values to those of PP fibers [39,40,41]. The reported tensile property values from the literature and the correspondence of their FTIR spectra to known spectra of different polymeric species enables the straightforward and robust identification of the fiber types in a CF bundle. 

**Figure 5:**
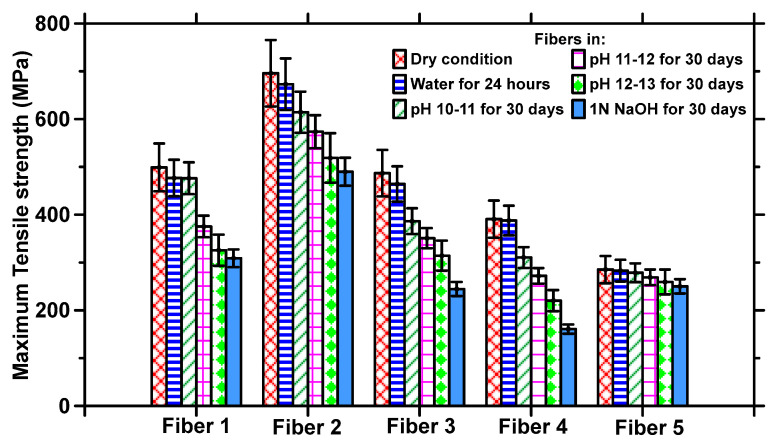
Effect of exposure conditions on the ultimate tensile strengths of selected fibers.

**Figure 6:**
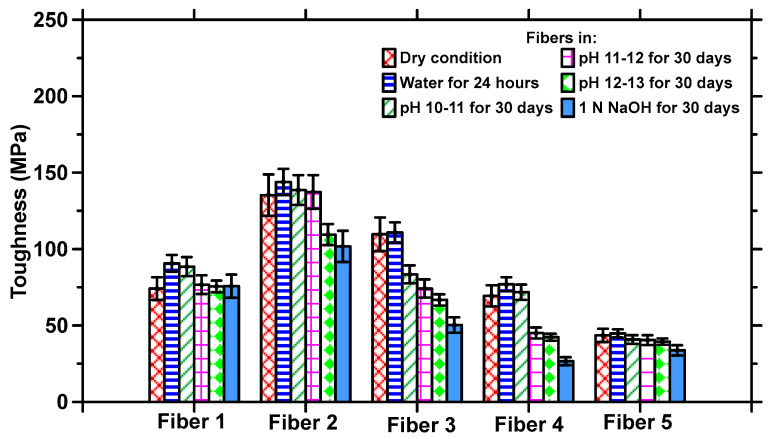
Effect of exposure conditions on the toughness of the selected fibers.

The high levels of moisture absorption of CFs influence their properties [43], which could impact their use in concrete. Here, the tensile properties of the fibers are used as an overall indicator of their behavior under sustained exposure to aggressive conditions. The tensile stress–strain responses of each type of fiber subjected to exposure to all the different conditions (water for 24 h, Ca(OH)_2_ solutions of pH 10–13 for 30 days, and 1 N NaOH for 30 days) are shown in Figure 4a–e. The residual tensile strengths of the fibers after the corresponding exposure conditions are shown in Figure 5, and the toughness values calculated from the stress–strain response based on a strain value corresponding to 50% of the peak stress in the post-peak zone are shown in Figure 6. 

Figure 4 and Figure 5 show that exposure to moisture for 24 h does not significantly influence the tensile strengths, as compared to those in the dry state, for CFs belonging to any of the dominant fiber types discussed earlier. Selected experiments on exposure to water at ambient temperature for longer durations did not result in appreciable changes in properties as compared to 24 h exposure, and hence they are not discussed further here. All polymeric materials absorb water, attributable to the hydrophilic groups present in them [44,45,46], resulting in some swelling and possible dissolution. The driving force for swelling is generally osmotic pressure, as a result of which the fiber molecules detach themselves from their neighbors, thereby contributing to an increase in elongation [46]. Nylon 6 and nylon 6,6 are moderately hydrophilic with a moisture regain of 4–5% under standard conditions and retrieval of 9% at an RH of 100% [47]. The degree of swelling and the resultant elongation is lower for hydrophobic PET fibers, which have a moisture regain of 0.1–0.4% in standard conditions and a 1% retrieval at 100% RH, thereby resulting in only a slight variation in tensile properties when subjected to moisture [48]. PP fibers are also relatively hydrophobic [49,50]. Under moisture exposure, the moderately hydrophilic nylon fibers showed slightly higher strength losses compared to the hydrophobic PET and PP fibers (see Figure 5), even though for all the fibers studied, the overall strength loss under moisture exposure is not very significant. 

The fibers were also immersed in Ca(OH)_2_ solutions of different pH levels [51,52] as well as 1 N NaOH solution for 30 d to simulate extreme exposure. Figure 4 shows that, in general, with increasing alkalinity, the tensile strength decreases and the elongation at peak stress increases. For the fibers of the nylon family (Figure 4a–c), the reduction in tensile strength as compared to the baseline state ranges from 25 to 50%, with the highest reduction being observed for nylon 6 fibers. Fibers 1, 3, and 4 (belonging to nylon and PET families) demonstrate a strength reduction of 50% or more between the pristine state and the alkaline conditions, while Fiber 2 (nylon 6,6/ZnO) demonstrates the highest pristine strength and the highest strength after degradation. This reduction in the strength of PET fibers in alkaline concrete environments has been previously reported [51], and the strength loss reported is comparable to those demonstrated at pH levels of 12–13 in this study. A comparison between the stress–strain curves in Figure 4 shows that the elongation at peak stress is higher when the samples are tested after immersion in water, depending on the fiber chemistry. Figure 5 and Figure 6 show that the strength and toughness loss for PP fibers under alkaline conditions chosen in this work are rather minimal (albeit with lower strength and toughness under pristine conditions). PP fibers are commonly used in concrete to mitigate shrinkage, and are reported to perform adequately in the presence of alkaline pore solution [51,53]. Microscopic and spectroscopic evidence of fiber degradation under different exposure conditions responsible for the aforementioned mechanical behavior are shown in a forthcoming section.

For all the fibers, 1 N NaOH exposure results in the highest strength reduction as well as in the largest elongation at peak, sometimes being twice as high as that in the dry state. For Fiber 1, the strength reduction is accompanied by a corresponding enhancement in strain until reaching 50% of the peak stress, resulting in comparable toughness values under all exposure conditions, as shown in Figure 6, while for Fibers 2–4, the strength reduction is more prominent than strain enhancement, leading to reduction in toughness under the chosen exposure conditions. For Fiber 5 (PP), the strength and toughness remain relatively unchanged following alkaline exposure, since these fibers hardly degrade, as demonstrated in the SEM images and FTIR spectra below. It is also well known that high temperature exposure accelerates hydrolysis and fiber depolymerization. However, since the objective of this work is to evaluate CF-reinforced concrete’s performance under ambient temperature, elevated temperature studies were not undertaken. 

### 3.2. Chemical and Microstructural Alterations of Fibers under Alkaline Exposure 

**Figure 7:**
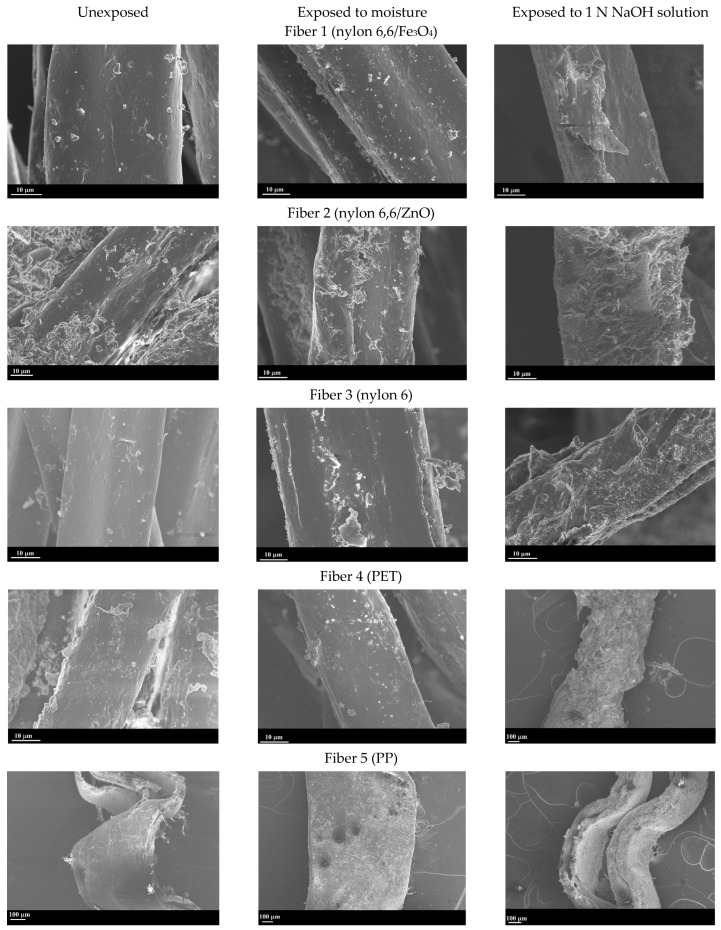
Representative SEM images showing morphological attributes of different types of fibers in the carpet fiber bale before and after exposure to moisture and 1 N NaOH.

Mechanical property changes under exposure to severe conditions can be ascribed to chemical changes occurring in the fibers, which influences their microstructure. Figure 7 shows the SEM images of all five fiber types in the bundle in the pristine state and after exposure to moisture and 1 N NaOH, depicting their morphological attributes. The fine particulates that are noticed in the images are calcium carbonate crystals, used as a filler material in carpet manufacturing. Exposure to moisture produces little to no changes in the morphology of the fibers, as can be noticed from Figure 7. When exposed to 1 N NaOH, Fibers 1–4 experience surface roughening and pitting, which is a result of the corrosion of the polymeric crystals and depolymerization. It is known that the alkaline hydrolysis of covalent bonds results in surface etching and cracking of nylon and PET fibers, as shown here, which is responsible for the property loss described earlier. However, even after exposure to extreme alkalinity (pH of ~14 for 1 N NaOH) for 30 d, fiber integrity is maintained in the nylon and PET fibers, as can be seen in Figure 7. PP fibers (Fiber 5) demonstrate little to no morphological changes when exposed to 1 N NaOH, substantiating the results from mechanical property evaluations described in the previous section. 

**Figure 8:**
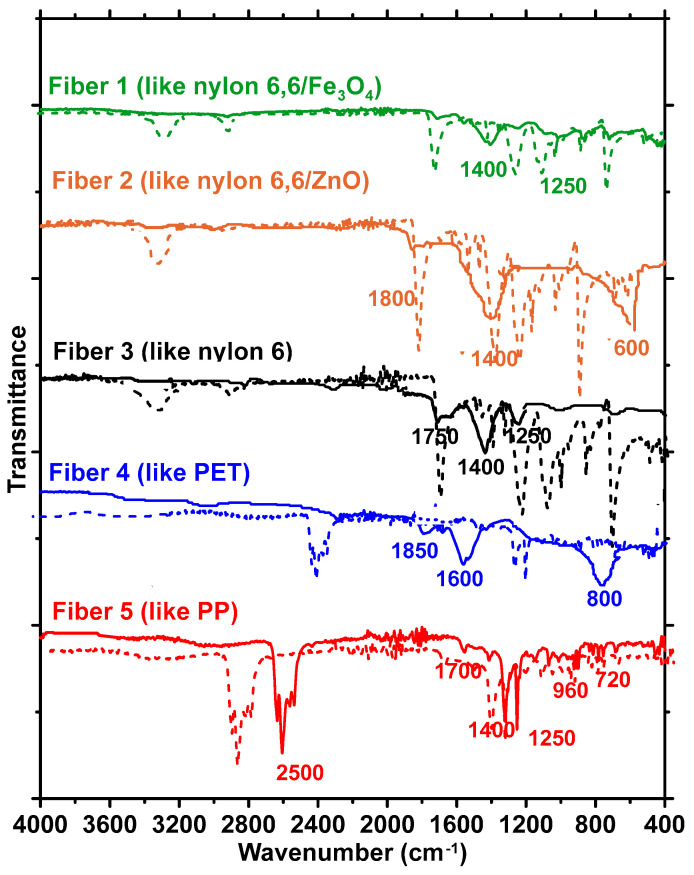
Representative FTIR spectra of different types of fibers in the carpet fiber bale: pristine fibers represented by dotted line, firm lines represent the samples after exposure to 1 N NaOH solution for 14 days.

Figure 8 shows the FTIR spectra of the fibers after exposure to 1 N NaOH solution. The dotted lines show the spectra for pristine fibers, which can be seen in Figure 2 as well. For the fibers belonging to the nylon family and PET, significant differences are noticed in the spectra before and after exposure. Peaks corresponding to several functional groups are absent in the spectra post-immersion. For the nylon 6,6/Fe_3_O_4_, nylon 6,6/ZnO, and nylon 6 fibers, the peaks at approximately 3300 cm^−1^, corresponding N-H stretching, are completely absent, whereas the peaks at 1680 cm^−1^ corresponding C-H bonds are shifted. For PET fibers, the peaks corresponding to C=O bonds (at 1720 cm^−1^) have disappeared completely. The PP fibers (Fiber 5) in the CF bundle are relatively unaffected by the 1 N NaOH exposure, which is consistent with the SEM images in Figure 7 as well as the tensile test results in Figure 4. Past studies have shown that even an exposure to 10% NaOH solution (2.5 N) at 60 °C for up to 4 h resulted in no significant difference in the FTIR spectra of PP fibers, and thus the alkaline environment in concrete can be considered not to degrade PP fibers [51]. 

**Figure 9:**
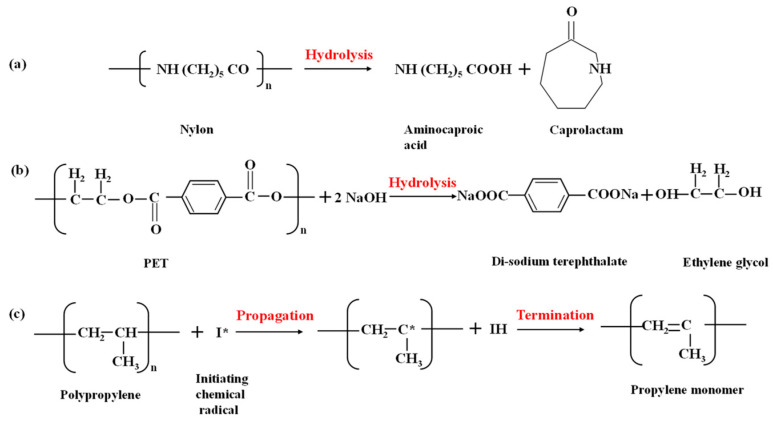
Mechanisms of depolymerization of (**a**) nylon [55,56], (**b**) PET [57], and (**c**) PP [54,58,59].

To shed more light on the mechanisms of fiber deterioration, their well-documented depolymerization mechanisms are shown in Figure 9 [54,55,56,57,58,59]. Polyamides such as nylon are almost unaffected by pure water (as shown using strength results and micrographs), but in the presence of strong acids or alkalis, they tend to depolymerize, as shown in Figure 9a [55,56]. The disappearance of N-H peaks at 3300 cm^−1^ can be considered an indication of amide hydrolysis occurring under basic conditions. Fiber depolymerization causes surface area reduction and surface defect creation (Figure 7), as well as progressive strength loss and an enhancement in elongation [60,61], as shown in Figure 4a–c. Solutions with pH ≥ 12 are noted to initiate strength loss in nylon fibers, with a precipitous decline in strength when the causticity of the exposure solution increases [48]. The PET fibers are also found to degrade under alkaline exposure, but contrary to nylon, the elongation at peak was not significantly increased, as shown in Figure 4d. The alkaline hydrolysis of PET (Figure 9b) leads to the formation of disodium terephthalate (with the FTIR spectrum demonstrating some of its characteristics peaks), and a more brittle fiber behavior [62]. It needs to be noted that alkaline hydrolysis at elevated temperatures is a commonly adopted method to recycle PET [63], where NaOH is used as a catalyst. Figure 4e and Figure 5 show that PP fibers present in the carpet fiber bale demonstrate very little strength loss or variations in the stress–strain response when subjected to the exposure conditions used in this study. For PP, the depolymerization reaction will start only if there some initiation agents like heat or chemical radicals are present [44,54,64,65,66], as shown in Figure 9c. Hence, it is not surprising that there are negligible microstructural changes or strength loss, as noticed in Figure 5 and Figure 7, irrespective of the exposure condition. 

### 3.3. Dimensional Changes in and Compressive Strengths of Mortar Mixtures under Alkaline Exposure

It can be noticed from the previous sections that the most severe exposure condition for the fibers, among those studied, is 1 N NaOH exposure. Hence, the dimensional change and compressive strength tests conducted on the mortars were limited to those exposed to 1 N NaOH. Mortar beams (mixtures MF0-MF5 shown in Table 2) were cured for 3 d in moisture and then immersed in 1 N NaOH solution for 25 d, and the changes in length were compared with those immersed in water for 28 d. Note that the CFs used in mortars and concretes are as-obtained fibers, with a combination of all five fiber types discussed in the previous sections, and hence the results are expected to be a composite response of these fiber types rather than the individual effects described in the previous sections. This also brings out another important factor with respect to the variability in the test results for concretes containing CFs. Depending on the relative fractions of the fiber types in a CF bale, the properties could also be different, and thus the analysis and discussions presented need to be contextualized within the limits of the fiber types encountered here. The results reported here pertain to the fiber bale under consideration, although the authors have taken sufficient care to ensure that the samples are representative as obtained from the carpet recycler. 

**Figure 10:**
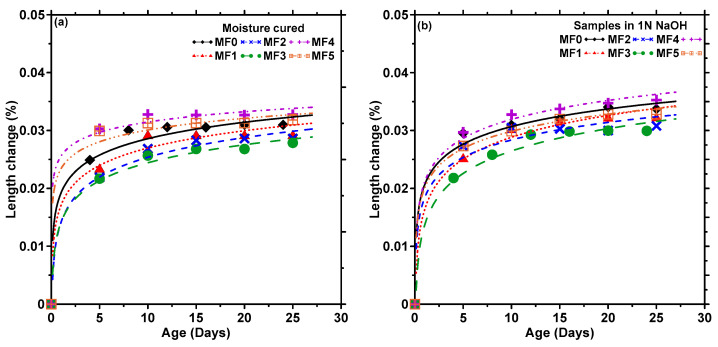
Dimensional changes in mortar beams: (**a**) during 28 d of moist curing and (**b**) after moist curing for 3 d followed by immersion in 1 N NaOH solution for 25 d. Average values from 3 or more companion specimens are reported.

Figure 10a,b show the change in length as a function of time for representative moisture-cured samples and those immersed in 1 N NaOH solution, respectively. In both cases, the unreinforced sample with 20% fly ash (MF1) shows a lower change in length than the control sample (MF0), as expected. The samples with 2.5% carpet fibers by volume (MF2 and MF3; without and with fly ash) are shown to have lower expansions than the control mixture; it is likely that water absorbed by the fibers leads to better hydration of cement and better pore filling in regions adjacent to the fibers (e.g., somewhat similar to internal curing effects), resulting in better dimensional stability. The crack-bridging capability of the carpet fibers also helps restrain crack propagation, consequently improving dimensional stability [67]. It is also conceivable that the penetration of NaOH into the mortar matrix could have resulted in fiber surface imperfections (see the micrographs in Figure 7), and coupled with enhanced cement hydration, both overall (due to higher alkalinity) and in the vicinity of the fibers, contributed to a better fiber–matrix bond. For the 5% fiber volume mixtures (MF4 and MF5), issues with fiber dispersion resulted in enhanced porosity of the material [17], and thereby somewhat higher expansion. Insufficient dispersion will enable easier debonding of the fibers from the matrix. This will decrease the crack-bridging ability and lead to higher expansion [68]. The samples exposed to 1 N NaOH show higher expansion than the moist-cured samples, even though under the experimental conditions evaluated the differences are rather minimal. While there is some fiber degradation (for most fiber types) under this exposure condition, as described earlier, they are not found to significantly influence the dimensional changes in mortars, likely attributed to some of the beneficial characteristics explained above. The results show that the chosen CF volumes can be tolerated in mortar specimens without any significant risk of dimensional instability.

**Figure 11:**
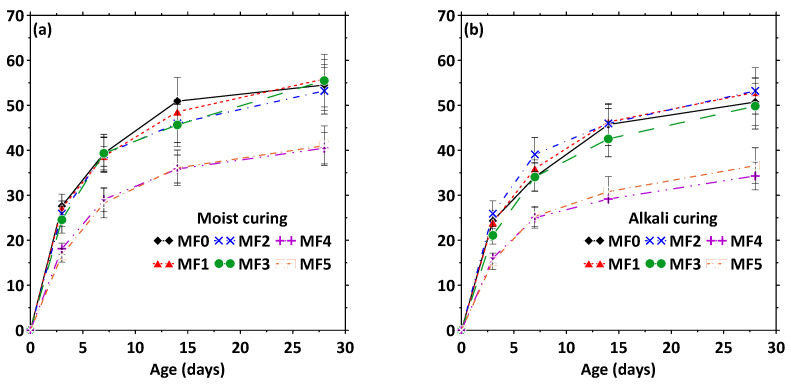
Compressive strength of CF concrete specimens: (**a**) moisture-cured for 28 d and (**b**) moisture-cured for 7 d and exposed to 1 N NaOH solution for 21 d. The standard deviations correspond to three companion samples tested.

The compressive strength of mortar mixtures made with 0%, 2.5%, and 5% CFs by volume and moisture-cured for 28 d or exposed to 1 N NaOH solution for 21 d after being moisture-cured for 7 d are shown in Figure 11a,b. Figure 11a shows that the use of 2.5% fibers by volume (mixtures MF2 and MF3) results in negligible strength loss under moist-curing conditions, whereas the incorporation of 5% fibers by volume (mixtures MF4 and MF5) results in an approximately 25% loss in compressive strength. Inefficient fiber dispersion, increased porosity, and the need for a higher w/b ratio are some of the important reasons for this strength loss [17]. Under exposure to 1 N NaOH solution, the trends for age-dependent compressive strengths are the same as those under moist-curing, but the strengths, especially at 28 d, are lower. A higher strength loss is noticed for the 5% fiber volume mixtures. The degradation of more fibers under an alkaline environment, along with the introduction of greater pore volumes due to fiber degradation (in addition to the already high porosity), in these specimens likely results in a more pronounced strength loss under exposure to severe alkalinity. 

### 3.4. Chloride Transport Behavior of Carpet-Fiber-Reinforced Concretes

**Figure 12:**
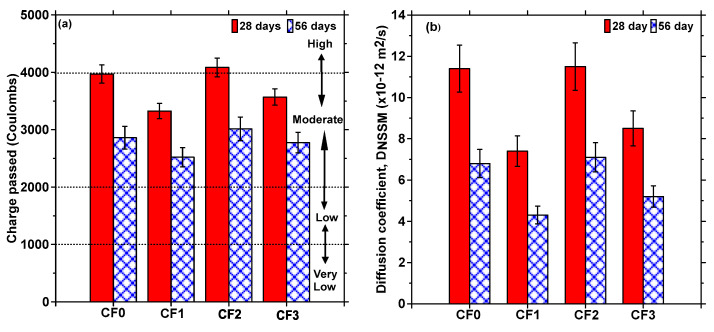
Chloride transport test results in terms of (**a**) charge passed during RCP test and (**b**) non-steady state migration coefficients for the concrete mixtures with and without carpet fibers. The standard deviations correspond to three companion samples tested.

The chloride transport studies were limited to concretes with 2.5% fiber volume, since this fiber volume fraction was the maximum limit that our implementation partner chose for use on slabs-on-grade construction based on the mechanical property results. Figure 12a depicts the total charge passed through the chosen mixtures during the rapid chloride permeability (RCP) test. At both ages, the mixtures containing fly ash demonstrate lower total charge passed, as expected [32,69,70]. The CF-reinforced mixtures, viz., CF2 and CF3, demonstrate comparable RCP values to their non-CF counterparts. This is not surprising, mostly because of the increase in the cementitious material content used when CFs are introduced into the mixture to attain comparable compressive strengths. Figure 12a also shows the classification range as per ASTM C 1202 [29], based on the total charge passed. The results indicate that, from a chloride transport standpoint (notwithstanding the inadequacies of the RCP test), the use of CF does not seem to alter the performance of concretes, within the limits of the carpet fiber volume used, or the volume recommended for use on slabs-on-grade and other moderately reinforced concrete structures. 

Non-steady state migration (NSSM) tests were also carried out to determine the electrically driven migration coefficients since they are reported to portray the chloride transport resistance of concretes more accurately [69,71,72]. Figure 12b shows the DNSSM values for the chosen mixtures. The plain concrete mixtures (without fly ash—CF0 and CF2) show similar NSSM values, and the ones containing fly ash (CF1 and CF3) show similar NSSM values, irrespective of the presence of CF, indicating that the likely enhancement in porosity due to the presence of a lower CF volume fraction is compensated by the presence of an increased amount of cementing/supplementary cementing materials. The presence of fly ash lowers the NSSM values, as expected. The variations in the NSSM values are higher than those for the RCP values, showing that this parameter is more sensitive to changes in mixture composition and thus the material’s microstructure. Overall, the NSSM values show that the incorporation of CFs in concrete at the dosages considered here does not impact the chloride transport resistance of concretes, and that with appropriate mixture-proportioning procedures (such as the use of fly ash or other partial cement replacement materials, better control of w/c, and means to ensure better fiber dispersion), better durability can be attained while making the concrete more sustainable through the utilization of a waste material that is otherwise landfilled. 

## 4. Summary, Conclusions, and Recommendations

Post-consumer carpets have a low recycling rate, and a majority of them end up in landfills. Sorting carpet face fibers is expensive, and not all fibers can be economically recycled, resulting in mixed carpet fibers being disposed—a majority of which are composed of nylon and PET. Thus, the utilization of carpet fibers as an ingredient in concrete is expected to be a significant contributor to reducing the landfilling of post-consumer carpets. While the use of 2.5% to 5% carpet fibers by volume in concrete does not significantly interfere with the concrete’s mechanical performance (provided appropriate proportioning procedures are undertaken), it is imperative to understand the durability of different types of fibers in a carpet bale when exposed to the alkaline environment in concrete, as well as the durability of concretes containing CFs. This study identified five different types of fibers—three belonging to the nylon family, along with PET and PP—in a typical carpet bale designated for landfilling. Nylon and PET were the dominant fiber types, which is typical. The fibers belonging to nylon and PET families showed significant strength reductions when exposed to alkaline conditions, with 1 N NaOH exposure for 30 d reducing the strength by ≥50%. Microscopic studies showed surface etching and roughening under alkaline exposure, while FTIR spectroscopy revealed the loss of several functional groups, which together explains the performance degradation. 

Since a typical fiber bale consists of fibers of all of the above types, it is important to recognize that the overall degree of deterioration (and thus the magnitude of property change) when CFs from a random bale are used in concrete will depend on the relative proportion of the different fiber types. The results reported here pertain to the chosen fiber bale (containing ~50% PET and ~40% nylon fibers, consistent with the most common types of commercial carpets). Mortar mixtures containing 2.5% fibers by volume showed comparable or lower dimensional changes compared to the control mixture under moist-curing conditions, while mixtures with a higher fiber volume showed increased expansion. The use of 2.5% fibers by volume resulted in comparable strength to control mixtures, but under alkaline exposure, the strengths were found to decrease, as expected. The chloride and moisture transport test results also indicated that the incorporation of 2.5% CFs by volume in concrete did not impact the concrete’s properties when compared to the control mixture. This study showed that, with appropriate mixture proportioning, including the use of fly ash, better control of w/c, and ensuring better fiber dispersion, 2.5% fibers by volume can be used in moderate-strength concretes without any major risk of performance degradation. 

Considering that ~2 million metric tons of carpet is landfilled in the U.S. annually, the face fibers amount to ~1 million metric tons. If 1/10th of the concrete produced in the U.S. (of the total ~600 million tons, of which >85% is of a strength ≤35 MPa) incorporates 25 kg of CFs per m^3^ (as recommended in this study), more than 0.6 million metric tons of CFs can be recycled and prevented from being landfilled. As described in this paper, comparable material performance and better environmental impacts are achieved through the use of post-consumer carpet fibers in concrete. Sustained efforts to modify concrete mixture proportions, develop and popularize methods and equipment to chop and disperse the fibers uniformly in concrete at an industrial scale, and implement cost-effective approaches to treat or functionalize the fibers to reduce water absorption are expected to enhance the acceptance of post-consumer carpet fibers in concrete. Synergy between landfill operators and concrete producers, testing and certification by independent agencies, and in-depth life-cycle and techno-economic analyses will likely provide a sustainable avenue for the diversion of post-consumer carpets from entering landfills or from being incinerated into buildings and infrastructure applications, thereby contributing to positive societal outcomes.

## Figures and Tables

**Table 1:** Chemical composition and physical properties of OPC and fly ash.

Binder Ingredients	Chemical Composition (% by Mass)							Specific Gravity
	SiO_2_	Al_2_O_3_	Fe_2_O_3_	CaO	MgO	SO_3_	LOI	
OPC (Type I/II)	20.98	3.82	3.82	63.54	1.82	2.84	1.27	3.15
Fly ash (FA)	54.93	20.44	4.57	9.93	2.81	0.86	0.99	2.28

**Table 2:** Mixture proportions for 1 m^3^ of mortar (OPC: cement; FA: fly ash; CF: carpet fiber).

Mix ID	% CF (by Vol)	OPC (Type I/II); kg	FA; kg	CF; kg	Sand; kg	Water; kg	Admixture; % by wt. of Binder	Water-to-Binder Ratio
MF0	0	413	0	0	1817	170	0.50	0.41
MF1	0	330	83	0	1791	170	0.50	0.41
MF2	2.5	567	0	23.8	1469	230	0.65	0.41
MF3	2.5	410	102	23.8	1534	210	0.65	0.41
MF4	5	567	0	47.5	1339	255	0.65	0.45
MF5	5	410	102	47.5	1418	230	0.65	0.45

**Table 3:** Mixture proportions for 1 m^3^ of concrete (OPC: cement; FA: fly ash; CF: carpet fiber; CA: coarse aggregate).

Mix ID	% CF (by Vol.)	OPC (Type I/II); kg	FA; kg	CF; kg	Sand; kg	CA; kg	Water; kg	Admixture; % by wt. of Binder	Water-to-Binder Ratio	28-Day Compressive Strength (MPa)
CF0	0	413	0	0	985	864	170	0.50	0.41	46.0
CF1	0	330	83	0	904	920	170	0.50	0.41	47.5
CF2	2.5	567	0	23.8	744	753	230	0.65	0.41	45.0
CF3	2.5	410	102	23.8	776	787	210	0.65	0.41	46.0

**Table 4:** Tensile properties of different types of fibers used in carpet manufacturing as obtained from literature. Figure 4 and Figure 5 provide the tested values for the different fiber types, which are further used (in addition to FTIR spectra) to identify and categorize the fibers in a CF bundle.

Fiber Type	Density (kg/m^3^)	Modulus of Elasticity (GPa)	Tensile Strength (MPa)	Elongation at Peak Load (%)
PP [39,40,41]	910	1.5–4.0	240–350	15–80
Nylon [36,42]	1140	2.0–5.2	402–902	15–45
Polyester (PET) [37,38]	1390	3.0–4.0	365–1096	15–50
Acrylic [1]	1180	2.0–3.0	208–490	20–45
Jute [1]	1300	10–30	390–800	1.5–1.8

## Data Availability

Data is available on request.

## References

[1] Pakravan H.R., Asgharian Jeddi A.A., Jamshidi M., Memarian F., Saghafi A.M. (2019). Properties of Recycled Carpet Fiber Reinforced Concrete. Use of Recycled Plastics in Eco-Efficient Concrete.

[2] Wang Y. (2006). Carpet Recycling Technologies. Recycling in Textiles.

[3] Peoples R. (2006). Carpet Stewardship in the United States—A Commitment to Sustainability. Recycling in Textiles.

[4] Peoples R. CARE 2015 Annual Report. https://carpetrecovery.org/wp-content/uploads/2014/04/CARE-2015-Annual-Report-FINAL-9-2-16-1.pdf.

[5] Thomassen M.A., Dalgaard R., Heijungs R., de Boer I. (2008). Attributional and Consequential LCA of Milk Production. Int. J. Life Cycle Assess..

[6] Sotayo A., Green S., Turvey G. (2015). Carpet Recycling: A Review of Recycled Carpets for Structural Composites. Environ. Technol. Innov..

[7] The Process That’s Saving Arizona’s Carpets from Landfill. https://www.nationalgeographic.com/environment/slideshow/partner-content-recovering-arizonas-rugs.

[8] Carpet America Recovery Effort. https://carpetrecovery.org/.

[9] Wang Y. (2010). Fiber and Textile Waste Utilization. Waste Biomass Valoriz..

[10] Wang Y., Zhang Y., Polk M.B., Kumar S., Muzzy J.D., Andrady A.L. (2004). Recycling of Carpet and Textile Fibers. Plastics and the Environment.

[11] Barbu B. A New Catalyst for Breaking Down Nylon 6. https://cen.acs.org/environment/recycling/new-catalyst-breaking-down-nylon/101/web/2023/12.

[12] Miraftab M., Lickfold A. (2008). Utilization of Carpet Waste in Reinforcement of Substandard Soils. J. Ind. Text..

[13] Fashandi H., Pakravan H.R., Latifi M. (2019). Application of Modified Carpet Waste Cuttings for Production of Eco-Efficient Lightweight Concrete. Constr. Build. Mater..

[14] Bolden J., Abu-Lebdeh T., Fini E. (2013). Utilization of recycled and waste materials in various construction applications. Am. J. Environ. Sci..

[15] Awal A.S.M.A., Mohammadhosseini H. (2016). Green Concrete Production Incorporating Waste Carpet Fiber and Palm Oil Fuel Ash. J. Clean. Prod..

[16] Pakravan H.R., Latifi M., Jamshidi M. (2017). Hybrid Short Fiber Reinforcement System in Concrete: A Review. Constr. Build. Mater..

[17] Simon A., Tripathi A., Surehali S., Neithalath N. (2023). Carpet Fiber Recycling in Regular-Use Concrete Mixtures and Associated Life Cycle Analysis. Waste Manag. Bull..

[18] Neithalath N. (2006). Analysis of Moisture Transport in Mortars and Concrete Using Sorption-Diffusion Approach. ACI Mater. J..

[19] (2020). C09 Committee. Test Method for Measurement of Rate of Absorption of Water by Hydraulic-Cement Concretes.

[20] Baldenebro-Lopez F.J. (2014). Influence of Continuous Plastic Fibers Reinforcement Arrangement in Concrete Strengthened. IOSR J. Eng..

[21] Gonzalez-Canche N.G., Flores-Johnson E.A., Cortes P., Carrillo J.G. (2018). Evaluation of Surface Treatments on 5052-H32 Aluminum Alloy for Enhancing the Interfacial Adhesion of Thermoplastic-Based Fiber Metal Laminates. Int. J. Adhes. Adhes..

[22] Fayemi O.E., Adekunle A.S., Ebenso E.E. (2016). A Sensor for the Determination of Lindane Using PANI/Zn, Fe(III) Oxides and Nylon 6,6/MWCNT/Zn, Fe(III) Oxides Nanofibers Modified Glassy Carbon Electrode. J. Nanomater..

[23] Ji Y., Yang X., Ji Z., Zhu L., Ma N., Chen D., Jia X., Tang J., Cao Y. (2020). DFT-Calculated IR Spectrum Amide I, II, and III Band Contributions of *N*-Methylacetamide Fine Components. ACS Omega.

[24] Du Y., George S.M. (2007). Molecular Layer Deposition of Nylon 66 Films Examined Using in Situ FTIR Spectroscopy. J. Phys. Chem. C.

[25] (2012). C01 Committee: Specification for Portland Cement.

[26] (2022). C09 Committee: Specification for Coal Ash and Raw or Calcined Natural Pozzolan for Use in Concrete.

[27] (2022). C09 Committee: Test Method for Length Change of Hardened Hydraulic-Cement Mortar and Concrete.

[28] (2001). C01 Committee: Test Method for Compressive Strength of Hydraulic Cement Mortars (Using 2-in. or [50mm] Cube Specimens).

[29] (2017). C09 Committee: Test Method for Electrical Indication of Concretes Ability to Resist Chloride Ion Penetration.

[30] (1999). Concrete, Mortar and Cement-Based Repair Materials: Chloride Migration Coefficient from Non-Steady-State Migration Experiments.

[31] Stanish K.D., Hooton R.D., Thomas M.D.A. (1997). Testing the Chloride Penetration Resistance of Concrete: A Literature Review.

[32] Cam H.T., Neithalath N. (2010). Moisture and Ionic Transport in Concretes Containing Coarse Limestone Powder. Cem. Concr. Compos..

[33] Neithalath N., Jain J. (2010). Relating Rapid Chloride Transport Parameters of Concretes to Microstructural Features Extracted from Electrical Impedance. Cem. Concr. Res..

[34] Vance K., Aguayo M., Dakhane A., Ravikumar D., Jain J., Neithalath N. (2014). Microstructural, Mechanical, and Durability Related Similarities in Concretes Based on OPC and Alkali-Activated Slag Binders. Int. J. Concr. Struct. Mater..

[35] Shahinian H., Cherukuri H., Mullany B. (2016). Evaluation of Fiber-Based Tools for Glass Polishing Using Experimental and Computational Approaches. Appl. Opt..

[36] Mahfuz H., Hasan M., Dhanak V., Beamson G., Stewart J., Rangari V., Wei X., Khabashesku V., Jeelani S. (2008). Reinforcement of Nylon 6 with Functionalized Silica Nanoparticles for Enhanced Tensile Strength and Modulus. Nanotechnology.

[37] Borg R.P., Baldacchino O., Ferrara L. (2016). Early Age Performance and Mechanical Characteristics of Recycled PET Fibre Reinforced Concrete. Constr. Build. Mater..

[38] Yildirim F.F., Avinc O., Yavas A. Elastic Polyesters. Proceedings of the Strutex 19th International Conference: Structure and Structural Mechanics of Textiles.

[39] Zhang M., Ma X., Liu Y., Ma J., Chen F., Zhang Q. (2019). High-Performance Electrospun POSS-(PMMA46)8/PVDF Hybrid Gel Polymer Electrolytes with PP Support for Li-Ion Batteries. Ionics.

[40] Wilson D. (2009). The Structure and Tensile Properties of Continuous Oxide Fibers. Handbook of Tensile Properties of Textile and Technical Fibres.

[41] Bunsell A.R. (2009). The Mechanical Behaviour of Small Diameter Silicon Carbide Fibres. Handbook of Tensile Properties of Textile and Technical Fibres.

[42] Xin H., Liu Y., Mosallam A.S., He J., Du A. (2017). Evaluation on Material Behaviors of Pultruded Glass Fiber Reinforced Polymer (GFRP) Laminates. Compos. Struct..

[43] Athijayamani A., Thiruchitrambalam M., Natarajan U., Pazhanivel B. (2009). Effect of Moisture Absorption on the Mechanical Properties of Randomly Oriented Natural Fibers/Polyester Hybrid Composite. Mater. Sci. Eng. A.

[44] Hanny R.A., Habib A.W. (2022). Polymers Degradation. Biomater. J..

[45] Boesel L.F., Reis R.L. (2008). A Review on the Polymer Properties of Hydrophilic, Partially Degradable and Bioactive Acrylic Cements (HDBC). Prog. Polym. Sci..

[46] Preston J.M., Nimkar M.V. (1949). Measuring the swelling of fibres in water. J. Text. Inst. Proc..

[47] Moody V., Needles H.L. (2004). Tufted Carpet: Textile Fibers, Dyes, Finishes and Processes.

[48] Needles H.L. (1986). Textile Fibers, Dyes, Finishes, and Processes: A Concise Guide.

[49] Sanjeevi S., Shanmugam V., Kumar S., Ganesan V., Sas G., Johnson D.J., Shanmugam M., Ayyanar A., Naresh K., Neisiany R.E. (2021). Effects of Water Absorption on the Mechanical Properties of Hybrid Natural Fibre/Phenol Formaldehyde Composites. Sci. Rep..

[50] Mihut C., Captain D.K., Gadala-Maria F., Amiridis M.D. (2004). Review: Recycling of nylon from carpet waste. Polym. Eng. Sci..

[51] Rostami R., Zarrebini M., Mandegari M., Sanginabadi K., Mostofinejad D., Abtahi S.M. (2019). The Effect of Concrete Alkalinity on Behavior of Reinforcing Polyester and Polypropylene Fibers with Similar Properties. Cem. Concr. Compos..

[52] Čorak I., Tarbuk A., Đorđević D., Višić K., Botteri L. (2022). Sustainable Alkaline Hydrolysis of Polyester Fabric at Low Temperature. Materials.

[53] Hannant D.J. (1983). Durability of Cement Sheets Reinforced with Fibrillated Polypropylene Networks. Mag. Concr. Res..

[54] Lilac W.D. (1999). Controlled Depolymerization of Polypropylene via Selective Partial Oxidation in a Supercritical Water Medium. Ph.D. Thesis.

[55] Bossert R.G., Croft R.C., Boord C.E. (1949). Hydrolysis of Nylon. J. Chem. Educ..

[56] Owen W.S. (1965). The Alkaline Hydrolysis of Nylons and Terylene. Microchim. Acta.

[57] Prorokova N., Chorev A., Kuzmin S., Vavilova S., Prorokov V. (2014). Chemical Method of Fibrous Materials Surface Activation on the Basis of Polyethylene Terephthalate (PET). Chem. Chem. Technol..

[58] Ebadi-Dehaghani H., Barikani M., Borhani S., Bolvardi B., Khonakdar H.A., Jafari S.H., Aarabi A. (2016). Biodegradation and Hydrolysis Studies on Polypropylene/Polylactide/Organo-Clay Nanocomposites. Polym. Bull..

[59] Park D.H., Kim M.S., Yang J.H., Lee D.J., Kim K.N., Hong B.K., Kim W.N. (2011). Effects of Compatibilizers and Hydrolysis on the Mechanical and Rheological Properties of Polypropylene/EPDM/Poly(Lactic Acid) Ternary Blends. Macromol. Res..

[60] He Y., Qian Z., Zhang H., Liu X. (2004). Alkaline Degradation Behavior of Polyesteramide Fibers: Surface Erosion. Colloid Polym. Sci..

[61] Das J., Halgeri A.B., Sahu V., Parikh P.A. (2007). Alkaline Hydrolysis of Poly(Ethylene Terephthalate) in Presence of a Phase Transfer Catalyst. Indian J. Chem. Technol..

[62] Silva D.A., Betioli A.M., Gleize P.J.P., Roman H.R., Gómez L., Ribeiro J. (2005). Degradation of Recycled PET Fibers in Portland Cement-Based Materials. Cem. Concr. Res..

[63] Park S.H., Kim S.H. (2014). Poly (Ethylene Terephthalate) Recycling for High Value Added Textiles. Fash. Text..

[64] Dong Q., Lele A.D., Zhao X., Li S., Cheng S., Wang Y., Cui M., Guo M., Brozena A.H., Lin Y. (2023). Depolymerization of Plastics by Means of Electrified Spatiotemporal Heating. Nature.

[65] Segre N., Tonella E., Joekes I. (1998). Evaluation of the Stability of Polypropylene Fibers in Environments Aggressive to Cement-Based Materials. Cem. Concr. Res..

[66] Akça K.R., Çakır Ö., İpek M. (2015). Properties of Polypropylene Fiber Reinforced Concrete Using Recycled Aggregates. Constr. Build. Mater..

[67] Ramezani M., Dehghani A., Sherif M.M. (2022). Carbon Nanotube Reinforced Cementitious Composites: A Comprehensive Review. Constr. Build. Mater..

[68] Ramezani M., Kim Y.H., Sun Z., Sherif M.M. (2022). Influence of Carbon Nanotubes on Properties of Cement Mortars Subjected to Alkali-Silica Reaction. Cem. Concr. Compos..

[69] Jain J.A., Neithalath N. (2010). Chloride Transport in Fly Ash and Glass Powder Modified Concretes—Influence of Test Methods on Microstructure. Cem. Concr. Compos..

[70] Poon C.S., Kou S.C., Lam L. (2006). Compressive Strength, Chloride Diffusivity and Pore Structure of High Performance Metakaolin and Silica Fume Concrete. Constr. Build. Mater..

[71] Ravikumar D., Neithalath N. (2013). Electrically Induced Chloride Ion Transport in Alkali Activated Slag Concretes and the Influence of Microstructure. Cem. Concr. Res..

[72] Aguayo M., Yang P., Vance K., Sant G., Neithalath N. (2014). Electrically Driven Chloride Ion Transport in Blended Binder Concretes: Insights from Experiments and Numerical Simulations. Cem. Concr. Res..

